# Cropland: Surplus or Deficit? From the Perspective of Meeting People’s Grain Requirement

**DOI:** 10.3390/foods12050964

**Published:** 2023-02-24

**Authors:** Yingnan Niu, Caixia Zhang, Gaodi Xie, Huan Niu

**Affiliations:** 1 Institute of Geographic Sciences and Natural Resources Research, Chinese Academy of Sciences, A11 Datun Road, Chaoyang District, Beijing 100101, China; 2 School of Urban Planning and Design, Peking University Shenzhen Graduate School, Shenzhen 518055, China; 3 College of Life Sciences, University of the Chinese Academy of Sciences, 19 A Yuquan Road, No.19, Shijingshan District, Beijing 100049, China

**Keywords:** cropland pressure, food security, spatiotemporal patterns, China

## Abstract

The quantity and quality of cropland plays an important role in ensuring food security. In order to explore spatiotemporal patterns of the extent to which cropland satisfies people’s grain need, we integrate multi-source heterogeneous data to investigate in which era, and in which region, the cultivated land can meet people’s food demands. It turns out that in the past 30 years, with the exception of the late 1980s, the amount of cropland could satisfy people’s grain needs at the nation scale. However, more than 10 provinces (municipality/autonomous region), mainly located in western China and southeast coastal areas, have been unable to meet the grain needs of local people. We projected the guarantee rate to the late 2020s. Our study concludes that the guarantee rate of cropland is estimated to be higher than 150% in China. Compared to 2019, except Beijing, Tianjin, Liaoning, Jilin, Ningxia, as well as Heilongjiang in the Sustainability scenario, and Shanghai in the Sustainability and the Equality scenarios, the guarantee rate of cultivated land will increase in every province (municipality/autonomous region) in 2030. This study has reference value for the study of China’s cultivated land protection system, as well as important significance for China’s sustainable development.

## 1. Introduction

Globally, factors such as population growth, higher incomes and urban lifestyles are driving changes in food demand and consumption, putting pressure on the quantity and quality of land resources [1,2]. Similarly, China now shares the same dilemma.

Since the reform and opening up in 1978, after 40 years of spectacular growth, China has become the world’s second-largest economy. The population has increased by almost 40 percent, from 987 million in 1980 to 1.412 billion in 2020 [3]. Meanwhile, a marked increase of urbanization rate, 19.39 percent in 1980 and 63.89 percent in 2020, has been seen in China over the last several decades [3]. At the same time, diets have changed, resulting in a higher proportion of non-starchy foods [4], with per capita consumption of meat, aquatic products, and eggs increasing by 0.23, 1.04, and 1.12 times, respectively, between 1990 and 2020 [3,5]. Moreover, the demand for animal product is projected to increase further in China, and livestock production will nearly double in the next few decades [6,7], which means higher requirement for feed grain. In addition to the change of diet structure, the reduction of cultivated land was along with the urbanization process [8], which brought great pressure to China’s food security.

A great deal of effort has been made in China to safeguard domestic food security. Grain output in China has increased steadily under a series of polices and measures implemented by the Chinese government and farmers, such as the delimitation of farmland protection red line and the construction of well-facilitated cropland, etc [9]. Grain output in 2020 increased 45 percent, 20 percent and 109 percent, respectively, when compared with that in 1980, 2000 and 2020 [3]. Among all kinds of grain output, rice, wheat, corn and tubers showed increasing trend in fluctuations, and the increase rates were 51 percent, 143 percent, 316 percent and 4 percent, respectively [3]. In terms of beans, a low peak appeared in the early 2010s, and then increased from mid 2010s, with a 28% increase rate from 1995 to 2020 [3]. While China has done well in domestic grain production, its ability to procure international grain resources has steadily improved. China’s grain imports have exceeded 100 million tons for seven consecutive years, reaching 164.53 million tons in 2021 [10]. 

Achievements in alleviating and eradicating hunger have increased in recent decades, but challenges remain. For example, land and water resources have never been more stressed and their accumulation is pushing the productive power of land and water systems to their ultimate limit. From 1990 to 2010, built-up land in China increased by 5.52 × 10^6^ hm^2^, which was mainly distributed in plains, rapidly expanded and densely populated regions, such as Huang-Huai-Hai Plain, the Yangtze River Delta, the Pearl River Delta, and the Sichuan Basin. Approximately 3.18 × 10^6^ hm^2^ of cropland were occupied for construction [8]. Moreover, owing to the impact of natural and human activities, different regions in China have suffered from soil erosion, which is threatening the stability of the agro-ecosystem and food security [11,12]. In addition, underground water depletion [13], irrigation water pollution and climate change [14] pose serious challenges to agriculture and food security [15]. More noteworthy is that food imports also face risks such as embargoes, rising food prices and poor food transportation in a complex international context.

Therefore, it is of great urgency to rethink deeply about China’s ability to ensure food security under these complex contexts. Many studies have concentrated upon the food security of China in the view of food production [16,17], food consumption [18], food trade [19], and the relationship between food supply and demand [9,20,21]. Cropland, the crucial factor for food production, has also been discussed all the time, from the quality to the quantity [22,23]. Nie analyzed the correlation between the quantity and quality of cultivated land and grain production and revealed the contribution of cultivated land to grain production and food security [24]. Sun et al. quantitatively analyzed the spatial-temporal coupling relationship between cultivated land change and grain yield increase in 12 northern provinces of China from 2000 to 2020 [25]. Geng studied the effect of the balance of cultivated land occupation and compensation on the grain production capacity of Jiangxi Province [26]. However, there are few studies on cultivated land demand that take into account population, per capita grain demand and grain yield per unit area. It is rare to explore the spatio-temporal demand for cropland and the extent to which cropland satisfies people’s needs in different regions in terms of meeting people’s nutritional needs, let alone the projections for the future. Based on this, this study aimed to: (1) figure out the amount of grain needed by people in China; (2) clarify how much of cropland is needed to feed people on the basis of part (1); (3) investigate the extent to which cropland can meet people’s grain need. We conducted this research from two spatial scales in five periods, that is, national and provincial scale in the late 1980s, 1990s, 2000s, 2010s, and 2020s. The results are of great significance for promoting sustainable development of China.

## 2. Methods and Materials

### 2.1. Method 

**Figure 1:**
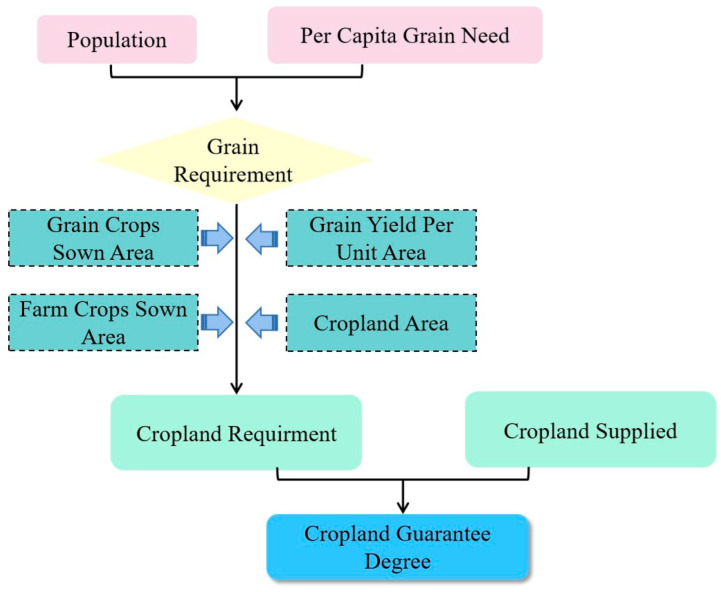
Technical flow chart of this study.

This study includes three steps for the analysis of the past and the future. First, we calculated people’s grain needs based on the population and per capita grain requirements. Then, by taking gain crops sown area, farm crops sown area, grain yield per unit area and cropland area into account, we obtain the cropland requirement. Finally, according to the cropland requirements, cropland guarantee degree was calculated (Figure 1). The following paragraphs illustrate the details of the calculation.

#### 2.1.1. Scenario Description

Projections of the level of cropland satisfying people’s grain need in the future are critical to enable a better understanding and anticipation of cropland’s bearing capacity. The climate projections and scenarios assessed by the IPCC (the Intergovernmental Panel on Climate Change, IPCC) based on SSP (the Shared Socioeconomic Pathway, SSP)- RCP (the Representative Concentration Pathway, RCP) framework, have furnished an exhaustive grasp of the restrictions and opportunities for policy action [27]. 

The Representative Concentration Pathway 2.6 represents scenarios that lead to quite low greenhouse gas concentrations. This is a scene of “peak and fall”. Its radiative forcing level first reached about 3.1 W/m^2^ by the middle of this century, and then returned to 2.6 W/m^2^ by 2100. In order to achieve such a level of radiative forcing, greenhouse gas emissions (as well as indirect emissions of air pollutants) should gradually decrease over time [28]. Under RCP 4.5, the total radiation forcing tends to be stable soon after 2100 and does not exceed the target level of long-term radiation forcing [29]. The Representative Concentration Pathway 3.4 denotes an intermediate mitigation effort pathway that lies between RCP 2.6 and RCP 4.5 [30].

The Shared Socioeconomic Pathway describes possible changes in various aspects of society in the 21st century, such as population, economy, technology, society, governance and environmental factors. The purpose is to promote a comprehensive analysis of future climate influence, vulnerability, adaptation and mitigation [31,32]. In SSP1, the world is gradually moving towards a more sustainable path, with a focus on development that respects environmental boundaries. SSP2 implies that the world is on an intermediate path, where social, economic and technological trends have not deviated significantly from the historical pattern. In SSP4, highly unequal social investment in human capital, coupled with growing inequality between economic opportunities and political power, has led to increasing inequality and stratification between and within countries. In this study, three climate models from CMIP6 (the Coupled Model Intercomparison Project Phase 6, CMIP6) were considered under the three SSPs in 2030, namely the Sustainability (SSP1-RCP2.6), “Middle of the Road” (SSP2-RCP4.5), and the Inequality (SSP4-RCP 3.4). These three scenarios describe the possible future world and represent different combinations of mitigation and adaptation challenges [30]. 

#### 2.1.2. Calculation of Grain Demand

In this study, people’s grain consumption was considered as the grain demand by people.

Ration and feed grain were two parts that people needed, and the calculation of grain demand is:

$${\mathrm{Grain}}_{requirement}=\left({\mathrm{Ration}}_{per\;capita}+\mathrm{Feed}\;{\mathrm{grain}}_{per\;capita}\right)\times Pop$$

where $Grain_{requirement}$ is the grain demand; $Ration_{per\;capita}$ and $Feed\;grain_{per\;capita}$ are ration and feed grain needed of each person, respectively; Pop is the population.

(1) Ration

The calculation of ration consumption in rural and urban areas is the same with previous study [9], and it can be described as follows:

$$\begin{array}{cc} Feed\;{\mathrm{grain}}_{all} & =Pork_{grain}+{\mathrm{Beef}}_{grain}+{\mathrm{Mutton}}_{grain}+Poultry\;{\mathrm{meat}}_{grain}+{\mathrm{Egg}}_{grain}\\ & +{\mathrm{Milk}}_{grain}+{\mathrm{Aquatic}\;\mathrm{product}}_{grain} \end{array}$$

where $Ration_{all}$ means the total amount of ration consumption in each province; $Ration_{urban\_per}$ and $Ration_{rural\_per}$ stand for per capita ration consumption in urban and rural areas, respectively; $Pop_{urban}$ and $Pop_{rural}$ mean the population in urban and rural areas. Details about population data can be seen in Section 2.2. The descriptions of per capita ration consumption are illustrated in Section 2.2.2.

(2) Feed Grain

The method of calculation of feed grain in rural and urban areas is similar to the previous study [9], which can be written as:

$$\begin{array}{cc} Feed\;grain_{all} & =Pork_{grain}+Beef_{grain}+Mutton_{grain}+Poultry\;meat_{grain}+Egg_{grain}\\ & +Milk_{grain}+{\mathrm{Aquatic}\;\mathrm{product}}_{grain} \end{array}$$

where $Feed\;grain_{all}$ represents total amount of feed grain needed, and $Pork_{grain}$, $Beef_{grain}$, $Mutton_{grain}$, $Chicken_{grain}$, $Egg_{grain}$, $Milk_{grain}$ and ${\mathrm{Aquatic}\;\mathrm{product}}_{grain}$ mean the amount of grain needed in the people’s consumption of pork, beef, mutton chicken, egg, milk and aquatic product, respectively. 

Here, we take the calculation of $Pork_{grain}$ as an example to illustrate the process:

$$Pork_{grain}=Pork_{grain\_urban}+Pork_{grain\_rural}$$

where $Pork_{grain}$ means the amount of grain needed in the people’s consumption of pork; $Pork_{grain\_urban}$ and $Pork_{grain\_rural}$ mean the amount of grain needed in the people’s consumption of pork in urban and rural areas, respectively.

$$\begin{array}{cc} Pork_{grain\_urban} & \\ & =(Pork_{per\_urban}\times Pop_{urban})\times \delta \times ({\mathrm{Rice}}_{\mathrm{pork}}+{\mathrm{Wheat}}_{\mathrm{pork}}+{\mathrm{Maize}}_{\mathrm{pork}}\\ & +{\mathrm{Soybean}}_{\mathrm{pork}}+{\mathrm{Tuber}}_{\mathrm{pork}}) \end{array}$$

where $Pork_{per\_urban}$ represents the amount of pork consumption for each person in urban areas; $Pop_{urban}$ represents the population in urban areas; δ represents forage required per unit of pork, which can be referred to in Table 1; ${\mathrm{Rice}}_{\mathrm{pork}},{\;\mathrm{Wheat}}_{\mathrm{pork}},{\;\mathrm{Maize}}_{\mathrm{pork}},{\;\mathrm{Soybean}}_{\mathrm{pork}}$ and ${\mathrm{Tuber}}_{\mathrm{pork}}$ represent the propotion of rice, wheat, maize, soybean and tuber in the forage, respectively, which can be referred to in Table 2.

The calculation of the amount of grain needed in the people’s consumption of pork in rural areas is the same as the calculation in urban areas.

However, there are some differences:

First, the change in feeding structure has been taken into account when calculating feed grain. The details are as follows:

Forage required per unit of product in 1989 and 1999 is based on the research results of Wang Minli and other researchers of the Institute of Agricultural Economy and Development, Chinese Academy of Agricultural Sciences [33]. With social and economic development, the livestock breeding structure in China has changed greatly, and breeding has gradually become large-scale. In the study of Xie [34], the feed required under the large-scale feeding for per unit product was illustrated. Therefore, the feed required per unit in the study of Xie et al. [34] was adopted to determine the forage required per unit of product in 2009, 2019 and 2030 in this study. The final results are shown in Table 1. 

Second, the proportion of edible parts of meat, aquatic products, milk and eggs and the loss of production and circulation are considered. The proportion of edible parts was 65.9%, 55.6%, 100% and 85% respectively, and the loss proportion was 15%, 34%, 6% and 10% respectively [34].

#### 2.1.3. Calculation of Cropland Requirement

The definition of cropland requirement is as below:

$$Cropland_{requirement}=Grain_{requirement}/\left(m\times n\times k\right)$$

where $Cropland_{requirement}$ is the amount of cropland needed; $Grain_{requirement}$ is the amount of grain needed by the people; *m* is the grain yield per unit area; *n* is the percentage of the area sown by grain crops in the area of cultivated land; *k* is multiple-crop index, which means the ratio of crop sown area to cultivated area. 

Data from 1989 to 2019 used in this part can be seen and calculated from Table 3. As for 2030, the data can be obtained from the following paragraphs [35]:

*m*: Due to the bottleneck of per unit yield potential of cultivated land, when the per unit yield level continues to increase and approaches the maximum per unit yield potential, the potential for per unit yield increase will gradually decrease. The function curve of exponential decay model can better reflect this change trend. This paper assumes that the external environment disturbance variable of crop growth is constant, that is, without considering crop improvement factors, we can use the grain yield data over the years (1997–2020) to build regression analysis models of every province, and the formula is as follow:

$$Y_{p}-Y_{t}=e^{-kt+b}$$

where $Y_{p}$ is the potential of average grain yield per unit area, which is 11,349.21 kg/hm^2^; $Y_{t}$ is the grain yield per unit area in year *t*. 

In this study, the values of *n* and *k* in 2030 originated from previous studies, which were 0.68 and 1.2 respectively. 

#### 2.1.4. Definition of Guarantee Level of Cropland

Guarantee level of cropland is defined as:

$$Rate_{cropland}=({\mathrm{Cropland}}_{\mathrm{supply}}/{\mathrm{Cropland}}_{\mathrm{required}})\times 100\%$$

where $Rate_{cropland}$ is the guarantee rate of cropland; ${\mathrm{Cropland}}_{\mathrm{supply}}$ is the quantity of cropland available; ${\mathrm{Cropland}}_{\mathrm{required}}$ is the quantity of cropland which needed by people.

### 2.2. Data Source

Six categories of data were applied in this study, including population, per capita grain requirement, cropland, per unit area grain yield, sown areas of farm crops, and sown areas of grain crops (Table 3), the details of which are presented in the following sections.

**Table 3:** Brief glance of data applied in this study.

Data	Time	Source
Population	1989, 1999,2009 and 2019	China Statistical Yearbook
	2030	https://dataguru.lu.se/app#worldpop, accessed on 2 September 2022
Per Capita Grain Requirement	1989, 1999, 2009 and 2019	China Statistical Yearbook
	2030	China Dietary Nutrition Guidelines 2016
Cropland	1990, 2000, 2010 and 2020	https://www.resdc.cn/, accessed on 8 October 2022
	2030	https://www.geosimulation.cn/China_SSP-RCP_1km.html, accessed on 8 October 2022
Per unit area grain yield	1989, 1999, 2009 and 2019	China Statistical Yearbook
Sown Areas of Farm Crops		
Sown Areas of Grain Crops		

#### 2.2.1. Population

The population in 1989, 1999, 2009 and 2019, as well as population in rural and urban areas in 2009 and 2019 was collected from China Statistical Yearbook 1990, China Statistical Yearbook 2000, China Statistical Yearbook 2010 and China Statistical Yearbook 2020 [3,5,36,37]. By reason of the lack of rural and urban population in 1989 and 1999, we calculated the urban and rural population in 1989 and 1999 based on the proportion of urban population in 1990 and 2000 [5,36]. 

Population data in 2030 under three scenarios were accessed from the high resolution data set for global future population developed with RCP (the Representative Concentration Pathway, RCP) and SSP (the Shared Socioeconomic Pathway, SSP) scenarios (https://dataguru.lu.se/app#worldpop, accessed on 2 September 2022) [38]. 

Population of each province from 1989 to 2030 can be seen from Table 4.

#### 2.2.2. Per Capita Grain Requirement

People’s needs for grain include ration and feed grain. Therefore, per capita food consumption is necessary in the calculation of ration and feed grain. The data for per capita food consumption in rural and urban area in 1989, 1999, 2009, and 2019 were obtained from China Statistical Yearbook 1990, China Statistical Yearbook 2000, China Statistical Yearbook 2010 and China Statistical Yearbook 2020 [3,5,36,37]. 

The data on per capita food consumption in rural and urban areas in 2030 were from the China dietary nutrition guidelines [39].

#### 2.2.3. Cropland Data

The cropland data for 1989, 1999, 2009 and 2019 were obtained from the 1-Km land remote sensing data in 1990, 2000, 2010 and 2020 (https://www.resdc.cn/, accessed on 8 October 2022).

Gridded 1km land use/land cover change projections of China under comprehensive SSP-RCP (the Shared Socioeconomic Pathway and the Representative Concentration Pathway, SSP-RCP) scenarios of 2030 were used to obtain the cropland for 2030 (https://www.geosimulation.cn/, accessed on 8 October 2022). And the amount of cropland in each province can be seen from Table 5.

#### 2.2.4. Per Unit Area Grain Yield of Cropland 

Grain yield per unit area of 1989, 1999, 2009 and 2019 were accessed from China Statistical Yearbook 1990, China Statistical Yearbook 2000, China Statistical Yearbook 2010 and China Statistical Yearbook 2020 [3,5,36,37].

Based on the average increasing rate of per unit area grain yield from 1989 to 2019, we calculated the grain yield per unit area of 2030.

## 3. Results

### 3.1. Nationwide

During the past 30-year period from 1989 to 2019, the amount of grain needed by people rose from 3.43 × 10^11^ kg to 4.11 × 10^11^ kg. Compared with 2019, the amount of grain needed by people is projected to increase, which would reach 4.14 × 10^11^ kg, 4.25 × 10^11^ kg and 4.12 × 10^11^ kg in 2030 under the Sustainability, the Middle Road and the Inequality scenarios (Figure 1).

**Figure 2:**
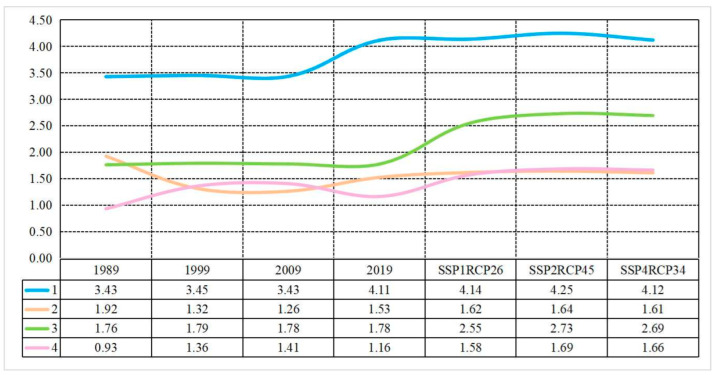
Amount of grain and cropland needed, cropland supplied and guarantee level of cropland in China at national scale, where 1 stands for the demand of grain, 10^11^ kg; 2 stands for the cropland demand, 10^8^ ha; 3 stands for cropland supplied, 10^8^ ha; 4 stands for guarantee rate of cropland, 100%.

If we convert grain demand to cropland demand, the amount of cropland needed in 1989, 1999, 2009, 2019 and the three scenarios (the Sustainability, the Middle Road and the Inequality) of 2030 is 1.92 × 10^8^ ha, 1.32 × 10^8^ ha, 1.26 × 10^8^ ha, 1.53 × 10^8^ ha, 1.62 × 10^8^ ha, 1.64 × 10^8^ ha and 1.61 × 10^8^ harespectively. However, the amount of cropland supplied is 1.76 × 10^8^ ha, 1.79 × 10^8^ ha, 1.78 × 10^8^ ha, 1.78 × 10^8^ ha, 2.55 × 10^8^ ha, 2.73 × 10^8^ ha and 2.69 × 10^8^ ha, respectively. This implies that the amount of cropland could satisfy people’s grain need except 1989. In addition, it can be seen that the ability of cropland to satisfy people’s grain needs increased from 1989 to 2009, while it decreased from 2009 to 2019. Under the three scenarios in 2030, the guarantee rate of cropland is projected to increase, with the Middle Road being the highest and the Sustainbility is expected to be the lowest (Figure 2).

### 3.2. Provincial Scale

#### 3.2.1. Amount of Grain Needed by People 

**Figure 3:**
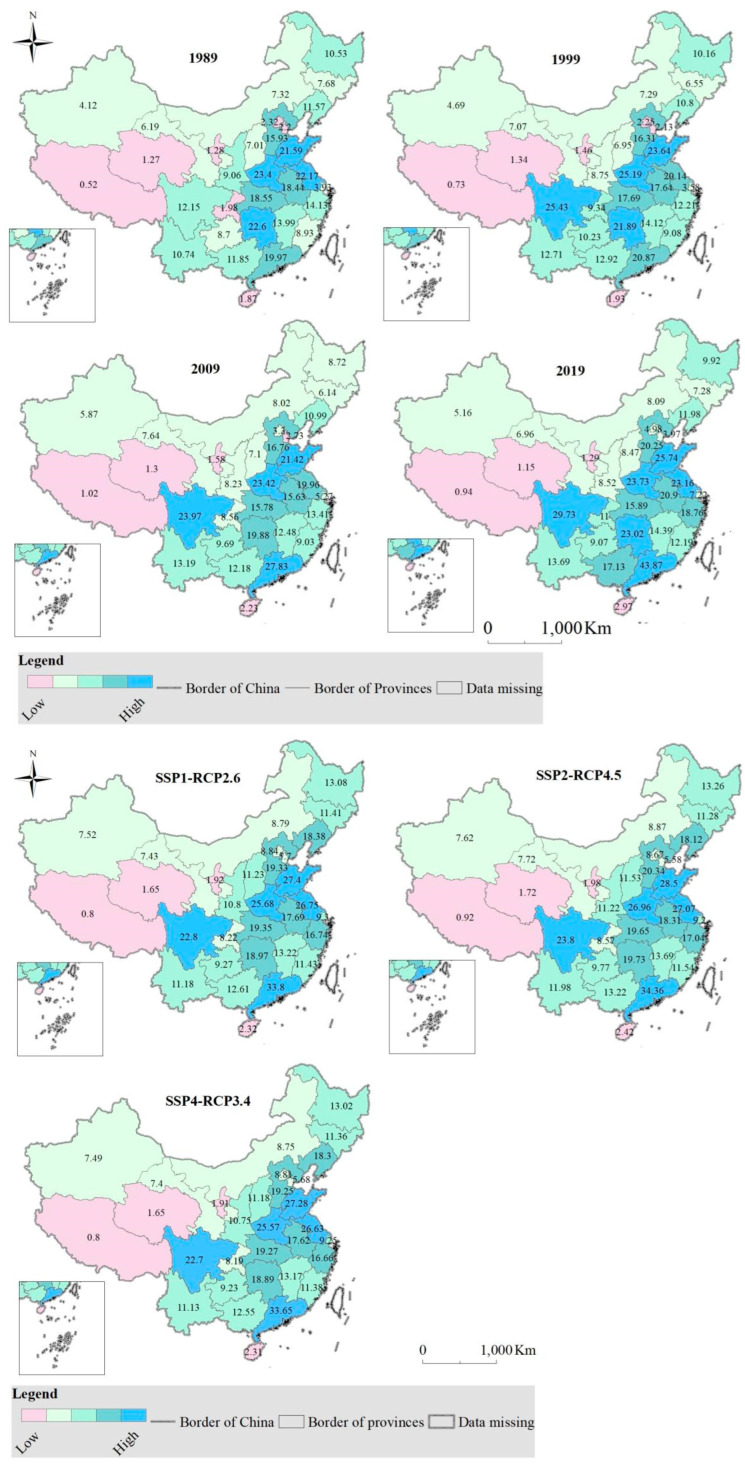
Grain required by people, and the value (unit: 10^9^ kg) in the map stands for the amount of grain required by people in each province.

To capture the regional heterogeneity of grain needed by people, we disaggregate grain consumption into five categories from low to high: Lowest (<3); Medium low (3≥ and <9); Low (9≥ and <15); Medium high (15≥ and <21); Highest (≥21). There are distinct patterns of the amount of grain needed by people (Figure 3): for example, relatively large shares of grain needed by people in Shandong, Henan, Sichuan, Jiangsu, Hunan and Guangdong. 

A look at demand patterns over time provides insight into the amount of grain needed by people (Figure 3). Over the past thirty years, Jilin, Heilongjiang, Hubei, Shaanxi and Qinghai have seen a declining trend in people’s grain demand, while the opposite has been true in other provinces. Under the Sustainability and the Inequality scenarios in 2030s, people’s grain demand in Hebei, Zhejiang, Anhui, Fujian, Jiangxi, Hunan, Guangdong, Guangxi, Hainan, Chongqing, Sichuan, Yunnan and Tibet is expected to decrease compared to the late 2010s, while the other provinces (municipality/autonomous region) show the opposite. With the exception of Hebei, the development of the Middle Road is the same as the above scenarios (Figure 3). 

#### 3.2.2. Cropland Needed by People

**Figure 4:**
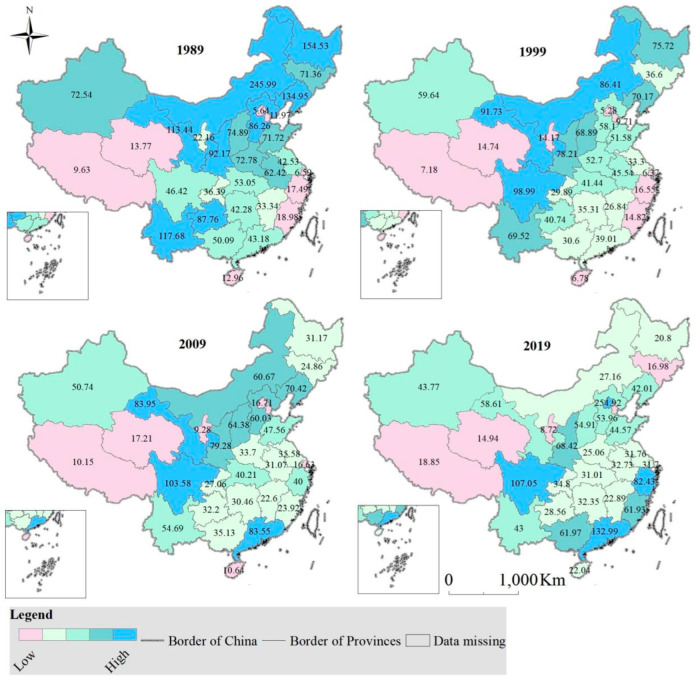

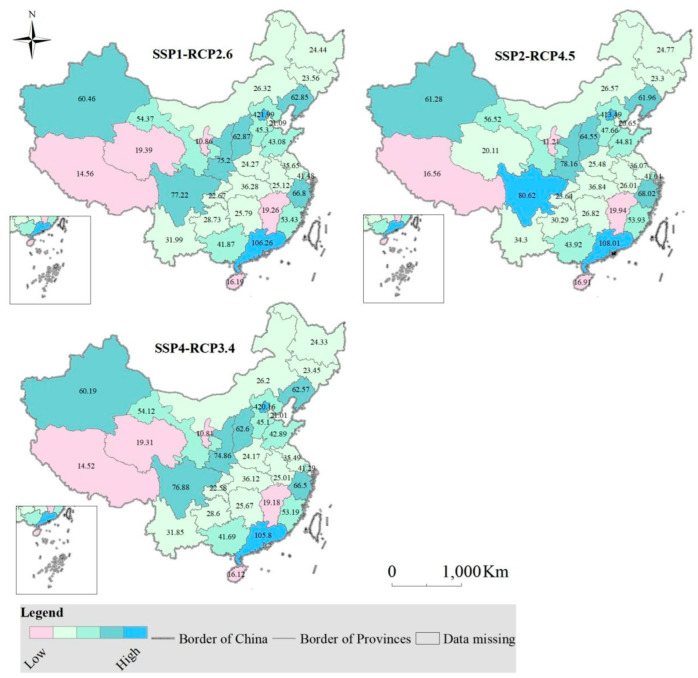
Cropland needed by people, and the value (unit: 10^5^ ha) in the map stands for the amount of cropland required by people in each province.

Delineating cropland by group is critical to understanding cropland demanding trends. Provinces in the western part of China led in cropland demand from 1989 to 2009. In 2019 and 2030, it can be seen that the southeastern coastal areas, from Huang Huai Hai Plain to Sichuan Basin areas, and northwest areas play major role in the demand for cropland (Figure 4). 

#### 3.2.3. Guarantee Rate of Cropland in the Past Few Years

**Figure 5:**
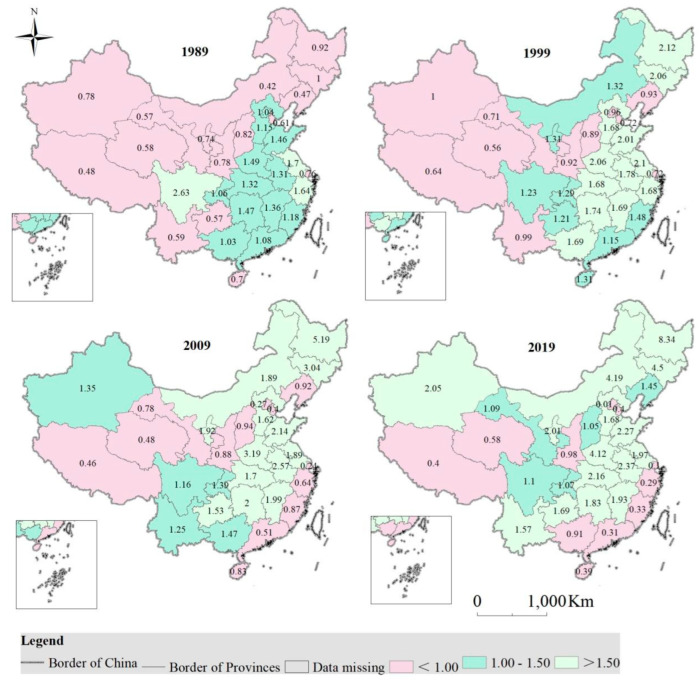
Guarantee rate of cropland in China from 1989 to 2019, and the value (unit: 100%) in the map stands for the guarantee rate in each province.

In the past thirty years, the guarantee rate of cultivated land in the other provinces (municipality/autonomous region) has declined, with the exception of Beijing, Tianjin, Shanghai, Zhejiang, Fujian, Guangdong, Guangxi, Hainan, Sichuan, Tibet, and Qinghai (Figure 5).

In 1989, cropland in Tianjin, Shanxi, Inner Mongolia, Liaoning, Jilin, Heilongjiang, Shanghai, Hainan, Guizhou, Yunnan, Tibet, Shaanxi, Gansu, Qinghai, Ningxia, and Xinjiang could not meet people’s grain needs. Cropland in Sichuan, Jiangsu and Fujian met people’s grain needs to a great extent. While the cropland of the remaining provinces (municipality/autonomous region) was able to satisfy people’s grain need basically.

In 1999, the cropland in Beijing, Tianjin, Shanxi, Liaoning, Shanghai, Yunnan, Tibet, Shaanxi, Gansu, Qinghai and Xinjiang was unable to meet people’s grain need. Cropland in Heilongjiang, Jilin, Hebei, Shandong, Henan, Hubei, Anhui, Jiangsu, Hunan, Jiangxi, Zhejiang and Guangxi met people’s grain need to a great extent. While the cropland in other provinces (municipality/autonomous region) was able to satisfy people’s grain need basically.

In 2009, cropland in Beijing, Tianjin, Shanxi, Liaoning, Shanghai, Zhejiang, Fu-jian, Guangdong, Hainan, Tibet, Shaanxi, Gansu and Qinghai could not meet people’s grain needs. Cropland in Inner Mongolia, Ningxia, Heilongjiang, Jilin, Hebei, Henan, Shandong, Jiangsu, Anhui, Hubei, Hunan, Jiangxi and Guizhou met people’s grain need to a great extent, while the cropland of the remaining provinces (municipality/autonomous region) was able to satisfy people’s grain need basically.

In 2019, the cropland in Beijing, Tianjin, Shanghai, Zhejiang, Fujian, Guangdong, Guangxi, Hainan, Tibet, Shaanxi and Qinghai was unable to meet people’s grain need. Cropland in Xinjiang, Inner Mongolia, Ningxia, Heilongjiang, Jilin, Hebei, Henan, Shandong, Jiangsu, Anhui, Hubei, Hunan, Jiangxi, Guizhou and Yunnan met people’s grain need to a great extent, while the cropland of the remaining provinces (municipality/autonomous region) was able to satisfy people’s grain need basically.

#### 3.2.4. Guarantee Rate of Cropland in the Future

**Figure 6:**
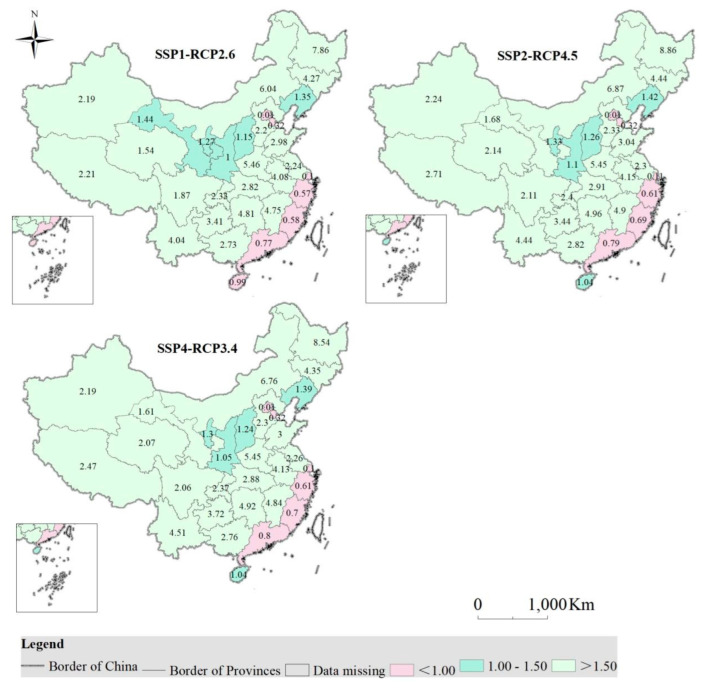
Guarantee rate of cropland in China in 2030 under three scenarios, and the value (unit: 100%) in the map stands for the guarantee rate of cropland in each province.

Under the Sustainability, the Middle Road and the Inequality scenarios, apart from Beijing, Tianjin, Shanghai, Jiangsu, Fujian, Guangdong, as well as Hainan in the Sustainability scenario, all provinces (municipality/autonomous region) are projected to achieve guarantee rate of cropland greater than 100% (Figure 6). 

With the exception of Beijing, Tianjin, Liaoning, Jilin, Ningxia as well as Heilongjiang in the sustainability scenario and Shanghai in the sustainability and equality scenario, the guaranteed rate pf cropland is expected to be higher in all provinces (municipality/autonomous region) compared to 2019 (Figure 6). 

## 4. Conclusions

In this study, we analyzed whether or not cropland in China could satisfy people’s grain needs across geographic and temporal scales. The following conclusions can be drawn:

(1) With the exception of 1989, the amount of cropland could meet people’s grain need. Moreover, the ability of cropland to satisfy people’s grain need increased from 1989 to 2009, while decreased from 2009 to 2019. Under the three scenarios in 2030, the guarantee rate of cropland is estimated to be higher than 150%. 

(2) From 1989 to 2019, the guarantee rate of cultivated land in other provinces (municipalities/autonomous regions) decreased except Beijing, Tianjin, Shanghai, Zhejiang, Fujian, Guangdong, Guangxi, Hainan, Sichuan, Tibet and Qinghai. Furthermore, more than 10 provinces (municipality/autonomous region), which are mainly located in western China and southeast coastal areas, were unable to satisfy the grain demand of local people. Compared to 2019, all provinces (municipalities/autonomous regions) except Beijing, Tianjin, Liaoning, Jilin, Ningxia, and Heilongjiang in the Sustainability scenario, and Shanghai in the Sustainability and the Equality scenarios, are projected to have a higher guarantee rate of cropland in 2030 than in 2019.

## 5. Discussion

The demand for cultivated land is the key content of this study. By comparing with the existing research, the demand for cultivated land calculated in 2030 (161 million hectares to 164 million hectares) is slightly higher than the previous research results (about 150 million hectares) [35], which is mainly related to the determination of the future population and the calculation method of the food required for nutritional needs.

From 1989 to 2009, under the comprehensive influence of population and per capita grain ration and feed grain consumption changes, the grain consumption demand of the Chinese population changed little, but due to technological progress, the unit yield of cultivated land increased, and the amount of cultivated land required decreased. As a result of urbanization and the project of returning farmland to forests, a large amount of cultivated land in China has been occupied [8,40]. However, the implementation of the policy of balancing the occupation and compensation of cultivated land has made the occupied cultivated land replenished [41,42], so the degree of China’s cultivated land to meet people’s food needs has been increasing. From 2009 to 2019, due to population growth, urbanization process and changes in the unit yield of cultivated land, the area of cultivated land required by Chinese residents for food consumption increased, while the actual existing cultivated land area changed little, and the final cultivated land satisfaction rate decreased. Under the three scenarios, China’s cultivated land satisfaction rate in 2030 is between 158% and 169%, showing a slightly tight balance. In addition, the future scenario is set on the basis of a reasonable dietary structure. If the current dietary structure is followed, the pressure on cultivated land in the future may be greater.

China is a vast country with large regional differences. The differences in natural conditions, diet structure, population development and urbanization process in different regions have led to differences in the cultivated land satisfaction rate in different regions of China. The cultivated land resources in western China are poor, and the problems of soil erosion, desertification and soil salinization are serious [43,44,45]. The local food production in these areas is facing serious challenges. Due to the rapid economic development in the eastern coastal areas, a large area of cultivated land has been occupied and food production has been hindered. 

China now is in a new era of food security. The decline in the area of cultivated land and low utilization efficiency have a very negative impact on the potential productivity of China’s cultivated land. Although the results of this study suggest that the amount of cultivated land in China will be more than 1.5 times of that needed to meet people’s grain needs by 2030, the marginalization of cultivated land in the process of urbanization, the non-agricultural transformation of cultivated land, and the transformation of agricultural cropping patterns to achieve economic benefits have potential impacts on China’s food production. Hence, under the background of food security, we need to reasonably use and control the cultivated land, strictly monitor the quantity of cultivated land, and improve the productivity of cultivated land. In addition, due to the geographical differences in population distribution, cultivated land quantity and cultivated land production potential, cultivated land protection in different regions of China faces various pressures. Therefore, under the strategic background of national food security, each region should actively explore and formulate cultivated land protection policies and agricultural production plans, that are suitable for itself and promote the sustainable development of China’s food production.

## Figures and Tables

**Table 1:** Forage required per unit of product from 1989 to 2030.

Year	Pork	Beef	Mutton	Chicken	Egg	Milk	Aquatic Product
1989	2.36	0.43	0.26	2.19	2.72	0.42	1.2
1999	2.09	0.49	0.54	1.62	1.69	0.39	1.2
2009	2.7	2.5	2.92	2.03	1.68	0.37	1.28
2019	2.7	2.5	2.92	2.03	1.68	0.37	1.28
2030	2.7	2.5	2.92	2.03	1.68	0.37	1.28

**Table 2:** Ratio of different types of grain needed in different forage, unit: %.

	Pork	Beef	Mutton	Poultry Meat	Egg	Milk	Aquatic Product
Rice	15.77	0	0	2.32	6.2	0	0
Wheat	6.70	5	5	2.33	2.7	0.7	7.08
Maize	43.57	26.25	26.25	50.82	40.40	31.40	23.39
Soybean	18.75	0	0	25	25	0	12.5
Tuber	15	0	0	0	0	0	0

**Table 4:** Population of each province from 1989 to 2030, unit: 10^7^.

		**Beijing**	**Tianjin**	**Hebei**	**Shanxi**	**Inner Mongolia**	**Liaoning**	**Jilin**	**Heilongjiang**
1989	Total	1.04	0.86	5.88	2.79	2.12	3.88	2.40	3.51
	Rural	0.23	0.27	4.76	2.02	1.36	1.90	1.38	1.85
	Urban	0.81	0.59	1.12	0.77	0.77	1.97	1.02	1.66
1999	Total	1.26	0.96	6.61	3.20	2.36	4.17	2.66	3.79
	Rural	0.28	0.27	4.89	2.09	1.35	1.91	1.34	1.84
	Urban	0.97	0.69	1.72	1.12	1.01	2.26	1.32	1.95
2009	Total	1.76	1.23	7.03	3.43	2.42	4.32	2.74	3.83
	Rural	0.26	0.27	4.01	1.85	1.13	1.71	1.28	1.70
	Urban	1.49	0.96	3.02	1.58	1.29	2.61	1.46	2.12
2019	Total	2.15	1.56	7.59	3.73	2.54	4.35	2.69	3.75
	Rural	0.29	0.26	3.22	1.51	0.93	1.39	1.12	1.47
	Urban	1.87	1.30	4.37	2.22	1.61	2.96	1.57	2.28
2030	SSP1RCP26	2.88	1.86	6.29	3.65	2.86	5.98	3.71	4.26
	SSP2RCP45	2.82	1.82	6.62	3.75	2.89	5.90	3.67	4.32
	SSP4RCP34	2.87	1.85	6.26	3.64	2.85	5.96	3.70	4.24
		**Shanghai**	**Jiangsu**	**Zhejiang**	**Anhui**	**Fujian**	**Jiangxi**	**Shandong**	**Henan**
1989	Total	1.28	6.54	4.21	5.47	2.90	3.70	8.16	8.23
	Rural	0.47	5.15	2.83	4.49	2.28	2.94	5.93	6.95
	Urban	0.80	1.39	1.38	0.98	0.62	0.75	2.23	1.28
1999	Total	1.47	7.21	4.48	6.24	3.32	4.23	8.88	9.39
	Rural	0.17	4.22	2.30	4.50	1.94	3.06	5.51	7.21
	Urban	1.30	2.99	2.18	1.73	1.38	1.17	3.38	2.18
2009	Total	1.92	7.73	5.18	6.13	3.63	4.43	9.47	9.49
	Rural	0.22	3.43	2.18	3.55	1.76	2.52	4.89	5.91
	Urban	1.70	4.30	3.00	2.58	1.86	1.91	4.58	3.58
2019	Total	2.43	8.07	5.85	6.37	3.97	4.67	10.07	9.64
	Rural	0.28	2.37	1.76	2.81	1.33	1.99	3.88	4.51
	Urban	2.14	5.70	4.10	3.55	2.64	2.68	6.19	5.13
2030	SSP1RCP26	3.03	8.71	5.45	5.76	3.72	4.30	8.92	8.36
	SSP2RCP45	2.99	8.81	5.55	5.96	3.76	4.46	9.28	8.77
	SSP4RCP34	3.01	8.67	5.42	5.73	3.71	4.29	8.88	8.32
		**Hubei**	**Hunan**	**Guangdong**	**Guangxi**	**Hainan**	**Chongqing**	**Sichuan**	**Guizhou**
1989	Total	5.26	6.01	6.03	4.15	0.64	1.47	9.23	3.17
	Rural	3.74	4.91	4.53	3.52	0.49	1.17	7.36	2.57
	Urban	1.52	1.10	1.50	0.63	0.15	0.30	1.87	0.60
1999	Total	5.94	6.53	7.27	4.71	0.76	3.08	8.55	3.71
	Rural	3.55	4.59	3.27	3.39	0.46	2.06	6.27	2.82
	Urban	2.39	1.94	4.00	1.33	0.31	1.02	2.28	0.89
2009	Total	5.72	6.41	9.64	4.86	0.86	2.86	8.19	3.80
	Rural	3.09	3.64	3.53	2.95	0.44	1.38	5.02	2.66
	Urban	2.63	2.77	6.11	1.90	0.42	1.47	3.17	1.14
2019	Total	5.93	6.92	11.52	4.96	0.95	3.12	8.38	3.62
	Rural	2.31	2.96	3.30	2.43	0.39	1.04	3.87	1.85
	Urban	3.62	3.96	8.23	2.53	0.56	2.09	4.51	1.78
2030	SSP1RCP26	6.30	6.17	11.00	4.10	0.76	2.68	7.42	3.02
	SSP2RCP45	6.39	6.42	11.18	4.30	0.79	2.79	7.75	3.18
	SSP4RCP34	6.27	6.15	10.95	4.08	0.75	2.67	7.39	3.00
		**Yunnan**	**Tibet**	**Shaanxi**	**Gansu**	**Qinghai**	**Ningxia**	**Xinjiang**	
1989	Total	3.65	0.22	3.19	2.17	0.44	0.46	1.45	
	Rural	3.11	0.19	2.51	1.69	0.32	0.34	0.99	
	Urban	0.54	0.03	0.69	0.48	0.12	0.12	0.46	
1999	Total	4.19	0.26	3.62	2.54	0.51	0.54	1.77	
	Rural	3.21	0.21	2.45	1.93	0.33	0.37	1.17	
	Urban	0.98	0.05	1.17	0.61	0.18	0.18	0.60	
2009	Total	4.57	0.29	3.77	2.64	0.56	0.63	2.16	
	Rural	3.02	0.22	2.13	1.77	0.32	0.34	1.30	
	Urban	1.55	0.07	1.64	0.86	0.23	0.29	0.86	
2019	Total	4.86	0.35	3.88	2.65	0.61	0.70	2.52	
	Rural	2.48	0.24	1.57	1.36	0.27	0.28	1.21	
	Urban	2.38	0.11	2.30	1.28	0.34	0.42	1.31	
2030	SSP1RCP26	3.64	0.26	3.51	2.42	0.54	0.62	2.45	
	SSP2RCP45	3.90	0.30	3.65	2.51	0.56	0.64	2.48	
	SSP4RCP34	3.62	0.26	3.50	2.41	0.54	0.62	2.44	

**Table 5:** Amount of cropland in each province from 1989 to 2030, unit: Km^2^.

					2030		
	1990	2000	2010	2020	SSP1RCP26	SSP2RCP45	SSP4RCP34
Beijing	5857	5048	4566	3670	5553	5703	5678
Tianjin	7280	6955	6729	5856	6827	6853	6756
Hebei	99,093	97,781	97,017	90,385	99,842	105,396	104,159
Shanxi	61,356	61,245	60,239	57715	72,065	79,091	78,135
Inner Mongolia	103,139	114,193	114,570	113,784	159,076	180,925	177,826
Liaoning	62,903	64,944	64,630	60,915	85,058	89,430	87,459
Jilin	71,115	75,279	75,543	76,470	100,566	104,619	102,457
Heilongjiang	141,886	160,293	161,880	173,578	192,124	216,636	208,699
Shanghai	4982	4556	3965	3326	4057	4450	4190
Jiangsu	72,336	69,947	67,256	62,485	79,789	81,969	80,678
Zhejiang	28,740	27,840	25,492	23,953	37,770	40,982	40,475
Anhui	81,515	80,883	79,858	77,586	102,471	104,234	103,691
Fujian	22,328	21,893	20,748	20,627	31,033	36,715	37,251
Jiangxi	45,477	45,389	45,071	44,199	91,506	94,368	93,310
Shandong	104,954	103,700	101,916	101,162	128,356	130,806	129,386
Henan	108,487	108,734	107,394	103,356	132,601	132,360	132,397
Hubei	70,215	69,651	68,460	66,951	102,260	105,645	104,571
Hunan	61,944	61,381	60,850	59,161	123,942	127,908	126,890
Guangdong	46,811	45,039	42,598	41,048	81,389	84,054	84,884
Guangxi	51,626	51,789	51,464	56,672	114,188	117,901	115,518
Hainan	9114	8907	8820	8680	15,948	16,916	16,776
Chongqing	38,687	38,507	37,732	37,411	52,830	54,446	53,645
Sichuan	121,932	121,447	120,198	117,841	144,255	163,013	158,982
Guizhou	49,794	49,444	49,326	48,279	98,097	98,848	106,962
Yunnan	69,128	69,070	68,436	67,459	129,368	141,898	144,170
Tibet	4638	4628	4620	7595	32,174	39,462	35,906
Shaanxi	71,760	71,748	69,900	66,843	75,442	83,093	78,901
Gansu	64,971	65,433	65,399	63,924	78,420	91,203	87,365
Qinghai	8002	8250	8286	8603	29,850	41,540	40,095
Ningxia	16,299	18,622	17,817	17,529	13,743	14,476	14,128
Xinjiang	56,629	59,396	68,736	89,931	132,624	135,156	132,484

## Data Availability

Data of this research are not shared.
